# *Cassia fistula* L. Fruit Extract Attenuates Renal and Cardiac Toxicities by Reducing Oxidative Stress and Inflammation in Fludrocortisone Acetate‐Treated Long Evans Rats

**DOI:** 10.1155/sci5/1517618

**Published:** 2026-11-01

**Authors:** Syeda Fatima-Tuz-Zahra, Sabrin Islam Khan, Tushar Emran, Zarin Tasnim Gias, Asim Kumar Bepari, Rezwana Ahmed, Preeti Jain, Hasan Mahmud Reza

**Affiliations:** ^1^ Department of Pharmaceutical Sciences North South University Dhaka Bangladesh northsouth.edu

**Keywords:** cardiac fibrosis, *Cassia fistula*, FCA, inflammation, oxidative stress, renal injury

## Abstract

Renal and cardiovascular diseases are major contributors to death worldwide. We investigated the therapeutic effect of *Cassia fistula* fruit extract (CFFE) on fludrocortisone acetate (FCA)–induced oxidative stress, renal toxicity, and cardiac fibrosis in rats. Long Evans rats were divided into different treatment groups, and biochemical tests were conducted on plasma, kidney, and heart homogenates to measure oxidative/antioxidative parameters and organ‐specific functional markers. The expression of proinflammatory and fibrotic genes in cardiac tissue was also measured. We observed that CFFE administration reduced the elevated levels of oxidative stress markers—malondialdehyde (MDA) and advanced oxidative protein products (AOPPs)—while enhancing the reduced antioxidant enzyme levels of catalase (CAT), superoxide dismutase (SOD), and glutathione (GSH) in FCA‐treated rats. Similarly, the CFFE treatment significantly reduced the increased plasma levels of cardiac troponin I (cTnI), uric acid, and creatinine in these rats. There was elevated expression of IL‐1β, IL‐6, TGF‐β1, and TNF‐α genes in the heart of the FCA rats, which was substantially downregulated by CFFE treatment. Histological investigations further revealed morphological abnormalities and fibrosis in the kidney and heart of the FCA rats, while CFFE treatment markedly ameliorated these signs. Our results suggest that CFFE prevents FCA‐induced oxidative stress, renal injury, and associated cardiac damage and fibrosis by enhancing the antioxidant defense system and controlling inflammation.

## 1. Introduction

The kidney and heart are functionally interconnected organs, and impairment in one system affects the other, leading to simultaneous failure of both organ systems [1]. Chronic kidney disease (CKD) has the capacity to trigger hypertension, and both CKD and hypertension gradually lead to cardiovascular diseases (CVDs) regardless of race and ethnicity [1, 2]. The association between CKD and hypertension is highly complex since hypertension can be a cause or consequence of impaired kidney function, and CKD patients carry a higher risk of premature death from cardiovascular events than from kidney disease [3]. Globally, in 2023, 788 million people aged 20 years and older were estimated to have CKD [4]. In 2017, approximately 9% of the population worldwide was affected by CKD, with a higher prevalence of 8%–16% reported in low‐income countries [5, 6]. On the other hand, hypertension, affecting nearly 30% of the adult population, is both a major risk factor and a contributing cause of cardiovascular conditions such as coronary heart disease, stroke, and heart failure [7–9]. CVDs are reported to account for 17.3 million deaths annually and are predicted to exceed 23.6 million deaths by 2030 [10, 11].

Earlier research indicates that the elevated levels of aldosterone can adversely affect the kidneys and heart by causing inflammation, oxidative stress, and fibrosis [11], leading to coronary artery disease, left ventricular hypertrophy, cardiomyopathy, and other heart ailments [12]. The aldosterone mineralocorticoid receptor (MR) complex triggers oxidative stress, activating NADPH oxidase and producing reactive oxygen species (ROS) that contribute to vascular inflammation and fibrosis [13]. Both oxidative and nitrosative stress damage lipids, proteins, and DNA in vascular endothelial cells, leading to endothelial dysfunction associated with inflammation and arterial stiffness [14].

Fludrocortisone acetate (FCA) is a well‐known synthetic adrenocortical steroid with strong mineralocorticoid activity that causes sodium and water retention in the kidneys [15, 16], thereby increasing cardiac workload and elevating blood pressure. Therefore, pharmacologically, FCA is indicated in the treatment of orthostatic hypotension [16–18]. In studies involving mice, FCA has been shown to enhance renal sodium reabsorption and induce pathological changes [19]. We previously demonstrated that FCA with 1% salt administration in uninephrectomized rats induced hypertension and caused cardiac and renal damage, including fibrosis [16, 20, 21]. Another study showed that intravenous administration of FCA, combined with a 1% salt‐supplemented diet in young adult Long Evans rats, induced oxidative injury, inflammatory responses, collagen buildup, and stiffness in heart and kidney tissues [22].

The therapeutic potential of various plant species has attracted considerable attention in the treatment of cardiac and renal disorders [23, 24]. *Cassia fistula* is a medicinal plant rich in metabolites that has been known to exhibit notable physiological effects on animals as well as humans, and several studies indicate that its fruits contain higher levels of mineral micronutrients compared with the other parts of the plant [25]. The presence of polyphenols, flavonoids, glycosides, proanthocyanidins, essential oils, tannins, and terpenoids [26] in *C. fistula* fruit extract has been associated with its antioxidant properties [27–30], along with potential benefits in managing CVDs, diabetes, stroke, and cancer [31, 32], as well as reducing oxidative stress and inflammation [33].

The antioxidant, hypolipidemic, and hepatoprotective potential of *C*. *fistula* fruits has been reported previously [33–35], along with other parts of the plant, such as bark and leaves [36]. However, the studies on the effects of CFFE on renal injury and cardiac damage are very limited. Therefore, the present study aimed to evaluate the potential of CFFE in ameliorating FCA‐induced renal and cardiac damage in rats. The key aspect of this study resides in the application of an FCA‐induced mineralocorticoid‐mediated cardiorenal injury model combined with a focused evaluation of *C. fistula* fruit‐specific pharmacological effects.

## 2. Materials and Methods

### 2.1. Drugs and Chemicals

FCA and amlodipine were purchased from Sigma‐Aldrich. Kits used to determine serum alkaline phosphatase (ALP), alanine aminotransferase (ALT), aspartate aminotransferase (AST), uric acid, and creatinine were purchased from Human Diagnostics, Germany. A cDNA synthesis kit from ABclonal, USA, was used to perform reverse transcription of the RNAs. All other chemicals and solvents used in the study were of analytical grade and obtained from Sigma or Merck.

### 2.2. Extract Preparation

Mature *C*. *fistula* fruits were obtained from Narsingdi, and they were authenticated (Accession No. DACB 135405) by the National Herbarium, Dhaka, Bangladesh. The collected fruits were washed and dried for several days. The dried fruits were ground into a powder and extracted with ethanol at room temperature for 7 days, with occasional shaking and stirring. The mixture was first filtered through a clean, white cotton cloth and finally through Whatman No. 1 filter paper. The extract was evaporated to dryness under reduced pressure by a rotary vacuum evaporator (EYELA N‐1200B, Tokyo, Japan) followed by overnight incubation at room temperature to ensure complete evaporation of residual solvents. The dried extract (4.5% w/w yield) was stored at 4°C in a refrigerator until used for analysis.

### 2.3. Experimental Animals

Thirty‐six male Long Evans rats (10 to 12 weeks old, 250 ± 10 g) were used in this experiment. They were obtained from the animal house of the Department of Pharmaceutical Sciences, North South University. The animals were maintained at a steady ambient temperature of 22 ± 2°C, a relative humidity of 55%, and a light‐dark cycle of 12/12 h. Rats were randomly grouped, and each group’s rats were placed in a distinct cage, with ad libitum access to standard chow pellets and water. Body weight and food and water intake were monitored regularly. The Institutional Animal Care and Use Committee (IACUC) at North South University approved the protocol, and we complied with the International Council for Laboratory Animal Science and the Council for International Organization of Medical Sciences (CIOMS/ICLAS, 2012) rules.

### 2.4. Preliminary Phytochemical Screening

The CFFE was subjected to preliminary phytochemical testing to detect the presence of various bioactive compounds. Wagner’s reagent was used for the detection of alkaloids, and FeCl_3_ was utilized to test the presence of phenolics and tannins. Salkowski’s test was used to test triterpenoids and steroids, whereas proteins and amino acids were detected by the biuret test and the ninhydrin test, respectively. The alkaline reagent test and the foam test were used to detect flavonoids and saponins, respectively. The Keller–Killiani test and the sulfuric acid test were carried out to detect glycosides and quinones, respectively. In each test, a 10% (w/v) solution of the ethanolic extract was used [37].

### 2.5. Determination of Total Phenolic Content (TPC)

The TPC in the extracts was determined as described previously [37]. Briefly, 0.3 mL of the extract was mixed with 1.2 mL of the 10% (v/v) Folin–Ciocalteu reagent solution and 1.5 mL of 7.5% (w/v) sodium carbonate solution. After incubating the mixture for 1 h, the absorbance was recorded at 765 nm. Gallic acid was used as a standard, and a concentration range of 50–500 μg/mL was used to obtain a standard curve (*Y* = 0.0011*x* + 0.0089, *R* ^2^ = 0.9997). The results were expressed as mg of gallic acid equivalents (GAEs) per gram of extract.

### 2.6. Determination of Total Flavonoid Content (TFC)

To determine the TFC of the extracts, 0.5 mL of the extract was mixed with 1.5 mL methanol, 0.1 mL of 1 M potassium acetate solution, 2.8 mL of distilled water, and 0.1 mL of 10% aluminum chloride solution [37]. This was followed by incubation at room temperature for 30 min, and the absorbance was measured at 415 nm. Standard solutions of quercetin with concentrations of 12.5–125 μg/mL were used to obtain a standard curve (*Y* = 0.006*x* + 0.0071, *R* ^2^ = 0.9995) and the results were expressed as mg of quercetin equivalents (QEs) per gram extract.

### 2.7. Experimental Design and Treatment

Experimental renal and cardiac toxicities were induced by administering FCA orally to the rats. To evaluate the in vivo effects of CFFE, the rats were randomly assigned to 6 groups (6 rats/group). The doses of FCA and CFFE were selected based on previous studies [16, 20, 21, 34, 38], and all treatments were carried out for 28 days. FCA, CFFE, and standard drug amlodipine besylate administrations were initiated from day 1. Group I, which served as the control, received only laboratory food and normal water. Group II (FCA), which represented the disease model, received FCA at 250 μg/kg/day, orally, along with 1% salt in drinking water. The treatment groups: FCA + CFFE100 mg, FCA + CFFE250 mg, and FCA + CFFE500 mg comprised FCA‐salt rats treated orally with CFFE at 100, 250, and 500 mg/kg/day, respectively. The standard Group (FCA + Amd) consisted of FCA‐salt rats administered amlodipine 10 mg/kg/day orally. The dose of amlodipine was selected based on previous studies [39, 40]. During the experimental period, the body weight and water intake of the animals were monitored daily. Following 28 days of treatment, all the rats were weighed, sacrificed under anesthesia (ketamine hydrochloride 100 mg/kg), and blood, kidneys, and heart were collected. The organs were weighed immediately; a portion from each organ was stored in neutral buffered formalin (pH 7.4) for histological analysis, and the remaining portion was stored at −20 °C for other studies. The collected blood was centrifuged at 5000 rpm, and the plasma was separated and stored at −20 °C for further analysis.

### 2.8. Assessment of Biochemical Markers

The biochemical tests for uric acid, creatinine, ALP, ALT, and AST levels were performed on plasma samples according to the protocols provided by the manufacturer (Human Lab, Germany).

### 2.9. Determination of cTnI

cTnI level was determined in plasma by the chemiluminescent microparticle immunoassay system using the manufacturer’s protocol (Human Cardiac Troponin I, Abcam, UK).

### 2.10. Tissue Homogenate Preparation

Heart and kidney tissues were homogenized with 10 volumes of phosphate buffer (pH 7.4) and centrifuged at 12,000 rpm for 30 min at 4 °C [41]. The collected supernatants were stored at −20 °C for further analysis.

### 2.11. Determination of Lipid Peroxidation as MDA

Thiobarbituric acid (TBA) 0.067 g was dissolved in 1 mL DMSO and 9 mL distilled water to prepare the TBA solution. Plasma sample (50 μL) or tissue homogenate (20 μL) was mixed with 50 or 80 μL of PBS solution, respectively. Then, 100 μL of glacial acetic acid was mixed with the sample solution, and the mixture was incubated for 15 min at room temperature. Following incubation, 200 μL TBA solution was added, and the mixture was kept in a water bath at 100 °C for 15 min, then cooled to room temperature. Two hundred (200) μL of this mixture was transferred to a 96‐well plate, and absorbance was measured at 532 nm. The extent of lipid peroxidation was expressed as MDA equivalents [42].

### 2.12. Determination of AOPP

AOPP levels were assessed as previously described [41]. Tissue homogenates (5 µL) or plasma (10 μL) were mixed with PBS to a final volume of 100 μL. Then, 50 μL potassium iodide and 50 μL acetic acid were added to this mixture, incubated at room temperature for 2 min, and the absorbance was recorded at 405 nm. Reaction mixtures without samples were used as blanks. AOPP was calculated from the chloramine‐T standard curve (0–100 nmol/mL) and expressed as nmol/gm tissue (heart and kidney) or nmol/mL plasma.

### 2.13. Determination of Myeloperoxidase (MPO)

We assessed MPO activity as described previously [43]. Briefly, 290 μL PBS was mixed with 10 μL tissue homogenate/plasma sample, and 3 μL of H_2_O_2_ and 3 μL of o‐dianisidine solution were added to the reaction mixture. To prepare the *o*‐dianisidine solution, 5 mL of concentrated HCl was mixed with 97 mL of distilled water, and then 20 mg of o‐dianisidine was added to 1 mL of the solution. The absorbance was measured at 450 nm for 5 min, and 1 unit of MPO activity was defined as the change in absorbance per minute.

### 2.14. Determination of SOD

Ten (10) μL tissue homogenate or plasma sample was added to 90 μL PBS in a 96‐well plate. Then 100 μL adrenaline was added to this mixture, and the absorbance was measured immediately at 450 nm [44]. Unit enzyme activity was determined from the amount of enzyme required to achieve 50% inhibitory effect of adrenaline in the reaction system [45].

### 2.15. Determination of GSH

In a 96‐well plate, 10 μL of plasma or tissue homogenate and 90 μL of PBS were taken. After adding 100 μL of DTNB (5,5‐dithiobis‐2‐nitrobenzoic acid, 0.04%) solution, the mixture was incubated at 4°C for 1 h, and the absorbance at 412 nm was measured immediately [42].

### 2.16. Determination of CAT

PBS (190 μL) was mixed with 10 μL of tissue homogenate or plasma sample in a 96‐well plate. We then added 100 μL of 0.06% H_2_O_2_ to the mixture. The absorbance of the mixture was measured at 240 nm at 0 and 1 min [41]. One unit of CAT activity was defined as a 0.01‐unit change in absorbance per minute.

### 2.17. Histopathological Studies

Heart and kidney tissues were fixed in 10% neutral‐buffered formalin and then treated with PBS, graded ethanol (50%, 70%, 90%, and 100%), and xylene and embedded in paraffin. The embedded tissue blocks were subjected to sectioning at a thickness of 5 μm. Later, tissue sections were in two changes of xylene for 5 min each and rehydrated sequentially through absolute ethanol, 95% ethanol, and 70% ethanol, followed by rinsing in PBS. The sections were then stained with hematoxylin for 3 min, followed by washing in tap water. Then the sections were dehydrated and stained in eosin for 3 min. Finally, they were mounted. On the other hand, for Sirius red staining, the sections were stained in picro‐Sirius red solution for an hour, rinsed in water, dehydrated, and mounted. All stained sections were observed under a microscope (Olympus DP12, Japan) and representative images were photographed at 20x magnification. For fibrosis scoring, ImageJ software was used. The area showing Sirius red‐positive staining was identified using a consistent thresholding procedure applied across the experimental groups. The percentage of Sirius red‐positive area relative to the total tissue area was calculated for each section and used as an estimate of collagen deposition.

### 2.18. Reverse Transcription and Polymerase Chain Reaction (RT‐PCR)

Heart tissues were collected from rats of different groups and washed in PBS. RNAs were isolated by homogenizing the tissues using TRIzol (Sigma‐Aldrich, USA) with a Dounce homogenizer. The yield and integrity of extracted RNA were assessed by measuring absorbance at 260 and 280 nm and by agarose gel (1%) electrophoresis. cDNAs were synthesized using the ProtoScript first‐strand cDNA synthesis kit (Promega, WI, USA) according to the manufacturer’s protocol. Semiquantitative PCR was performed with specific primer pairs and Taq polymerase (NEB, USA) using a TaKaRa PCR Thermal Cycler (TP650, TaKaRa, Japan). The reaction conditions used in PCR were as follows: initial denaturation at 94°C for 5 min, 30 cycles of denaturation at 94°C for 30 s, annealing at the appropriate temperature (Table 1) for 30 s, extension at 72°C for 30 s, and final extension at 72°C for 7 min. Finally, the gene expression was analyzed using agarose gel electrophoresis. ImageJ was used to quantify gene expression levels.

**TABLE 1:** Sequences of the primers used in this experiment.

Gene	Type	Sequence	Annealing temperature
GAPDH	Forward	5ʹ‐CTTGCCGTGGGTAGAGTCAT‐3ʹ	F‐ 60.5°C
	Reverse	5ʹ‐AGACAGCCGCATCTTCTTGT‐3ʹ	R‐ 58.4°C

IL‐1β	Forward	5ʹ‐AGACCTGACTTGGCAGAGA‐3ʹ	F‐ 57.3°C
	Reverse	5ʹ‐GCAATGGTCGGGACATAGTT‐3ʹ	R‐ 58.4°C

IL‐6	Forward	5ʹ‐CCCACCAGGAACGAAAGTCA‐3ʹ	F‐ 60.5°C
	Reverse	5ʹ‐GGCAACTGGCTGGAAGTCTC‐3	R‐ 63.3°C

TGF‐β1	Forward	5ʹ‐AATTCCTGGCGTTACCT‐3ʹ	F‐ 50°C
	Reverse	5ʹ‐CCTGTATTCCGTCTCCTT‐3ʹ	R‐ 53.9°C

TNF‐α	Forward	5ʹ‐TGTGGGTGAGGAGCACATAG‐3ʹ	F‐ 60.5°C
	Reverse	5ʹ‐GTCGTAGCAAACCACCAAGC‐3ʹ	R‐ 60.5°C

### 2.19. Statistical Analysis

All values are defined as mean ± standard deviation (SD). The results were analyzed by one‐way ANOVA followed by post hoc test using GraphPad Prism 8 software. Statistical significance was considered when *p* value was ≤ 0.05 in all cases. Comparisons between disease and treatment groups were conducted using an unpaired two‐tailed Student′s *t*‐test [46]. To minimize selection bias, the tissue sections were coded and evaluated under blinded conditions.

## 3. Results

### 3.1. Phytochemicals Analysis

Qualitative phytochemical screening of CFFE revealed the presence of various secondary metabolites, which included alkaloids, phenolics, tannins, flavonoids, saponins, glycosides, quinones, proteins, and amino acids. Quantitative analysis showed that the TPC was 58.09 ± 7.39 mg GAE/g extract and the TFC was 25.05 ± 6.92 mg QE/g extract.

### 3.2. Effects of CFFE on Body Weight and Organ Wet Weight

The body weight of each rat was measured daily during the experimental period. Compared with the control group, FCA‐treated rats showed a slight increase in body weight, whereas rats treated with the high dose of CFFE (500 mg/kg) and amlodipine exhibited a modest reduction in body weight (Figure 1a). Other groups showed almost similar weights.

**FIGURE 1:**
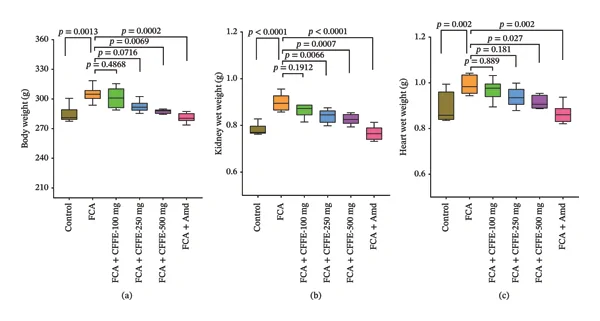
Effect of CFFE on body weight (a), kidney wet weight (b), and heart wet weight (c) of rats (*n* = 6). Statistical analysis was performed using one‐way ANOVA followed by the Newman–Keuls post hoc test. Statistical significance is considered at *p* ≤ 0.05, and ns indicates not significant.

Similarly, the kidney wet weight was slightly increased in FCA‐treated rats, which was mitigated by medium (250 mg/kg) and high (500 mg/kg) doses of CFFE and amlodipine (Figure 1b). In the case of heart wet weight, similar effects were observed (Figure 1c).

### 3.3. Effect of CFFE on cTnI, Uric Acid and Creatinine

cTnI is the primary indicator used to detect cardiac muscle damage. The cTnI level in all six groups was measured. The level of cTnI was significantly elevated in FCA‐treated rats as compared to the control rats (Figure 2a, *p* ≤ 0.05). However, with the administration of the CFFE and amlodipine, there was a marked decrease in the cTnI level in the plasma (Figure 2a).

**FIGURE 2:**
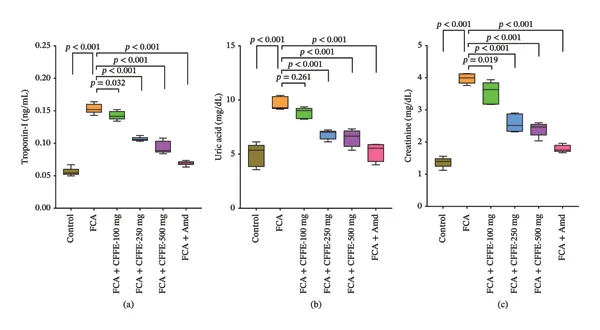
Effect of CFFE on cardiac troponin‐I (a), uric acid (b), and creatinine (c) concentration in plasma of rats (*n* = 6). Statistical analysis was performed using one‐way ANOVA followed by the Newman–Keuls post hoc test. Statistical significance is considered at *p* ≤ 0.05, and ns indicates not significant.

Uric acid and creatinine levels in plasma, on the other hand, are indicative of renal function. We measured both uric acid and creatinine levels in rats’ plasma. The results revealed that FCA‐treated rats had a substantial increase in uric acid levels compared with control rats (Figure 2b, *p* ≤ 0.05), and this increase was reduced in CFFE‐administered rats. Plasma creatinine levels in FCA‐treated rats also showed a marked elevation compared with the control group, but CFFE treatment reversed this level significantly (Figure 2c, *p* ≤ 0.05). Furthermore, compared with lower doses (100 & 250 mg/kg), the high dose (500 mg/kg) of CFFE produced a more pronounced response (Figure 2a–c). The standard drug amlodipine exhibited the highest effect.

### 3.4. Effect of CFFE on Serum ALT, AST, and ALP

The effects of CFFE supplementation on liver enzymes were examined. The levels of ALT, AST, and ALP were considerably higher in the FCA‐treated rats compared to the control rats (Figure 3a–c). Supplementation with CFFE showed a dose‐dependent improvement in plasma ALT, AST, and ALP levels. Moreover, CFFE showed a stronger effect in lowering ALP and ALT compared to AST in plasma (Figure 3a–c, *p* ≤ 0.05). The effect of the standard drug amlodipine was more pronounced.

**FIGURE 3:**
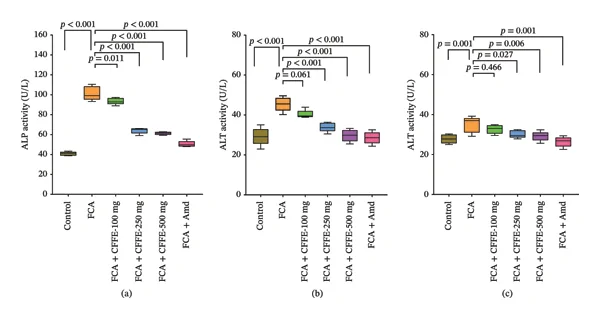
Effect of CFFE on ALP (a), ALT (b), and AST (c) levels in plasma. Statistical analysis was performed using one‐way ANOVA followed by the Newman–Keuls post hoc test. Statistical significance is considered at *p* ≤ 0.05, and ns indicates not significant.

### 3.5. Effect of CFFE on Oxidative Stress Parameters in Plasma, Kidney, and Heart Tissues

Compared with the control group, FCA‐treated rats showed significantly higher concentrations (*p* ≤ 0.05) of MDA, an oxidative stress marker, in kidney and heart homogenates and in serum (Figure 4a,c,e, respectively), which were markedly attenuated by CFFE administration. Furthermore, AOPP levels were also markedly elevated in the kidney, heart, and plasma of FCA‐treated rats, and were significantly reduced by CFFE administration in a dose‐dependent manner (Figure 4b,d,f, respectively). The highest reduction was observed with amlodipine.

**FIGURE 4:**
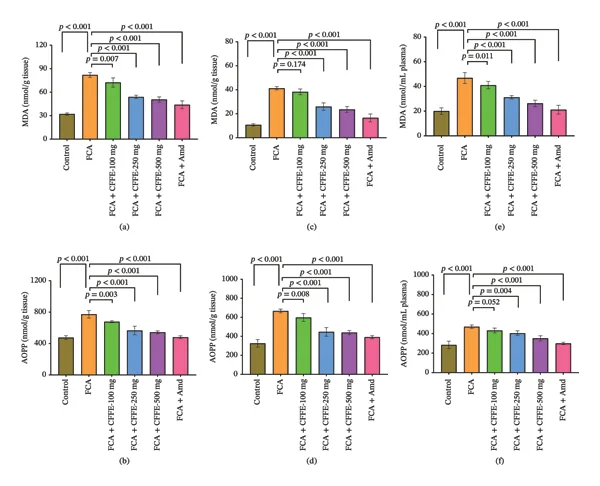
Effect of CFFE on MDA (a, c, e) and AOPP (b, d, f) concentrations in kidney, heart and plasma. Statistical analysis was performed using one‐way ANOVA followed by the Newman–Keuls post hoc test. Statistical significance is considered at *p* ≤ 0.05, and ns indicates not significant.

### 3.6. Effect of CFFE on MPO in Plasma, Kidney, and Heart Tissues

MPO serves as a marker of tissue inflammation and fibrosis; therefore, its activity was assessed in the kidney, heart, and plasma. The FCA group showed a significantly higher level of MPO activity than the control group (Figure 5a–c), which was reduced by CFFE administration. A significantly marked reduction was observed with the high dose of CFFE (500 mg/kg) as well as with amlodipine.

**FIGURE 5:**
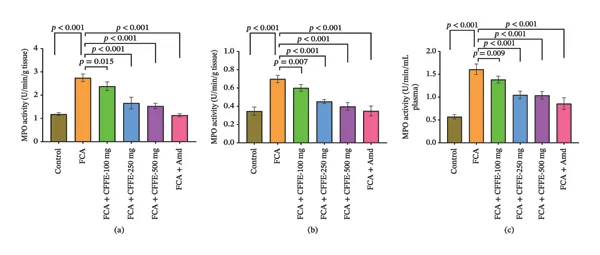
Effect of CFFE on MPO activity in kidney (a), heart (b), and plasma (c). Statistical analysis was performed using one‐way ANOVA followed by the Newman–Keuls post hoc test. Statistical significance is considered at *p* ≤ 0.05, and ns indicates not significant.

### 3.7. Effect of CFFE on Antioxidant Activity in Plasma, Kidney, and Heart Tissues

Compared with the untreated control group, FCA‐treated rats showed a significant reduction in the activities of antioxidant enzymes SOD, GSH, and CAT in the kidney (Figure 6a–c), heart (Figure 6d–f), and plasma (Figure 6g–i). CFFE administration exhibited a dose‐dependent increase in the SOD, GSH, and CAT activities. The effects produced by amlodipine were comparable to those observed with the high dose of CFFE (500 mg/kg).

**FIGURE 6:**
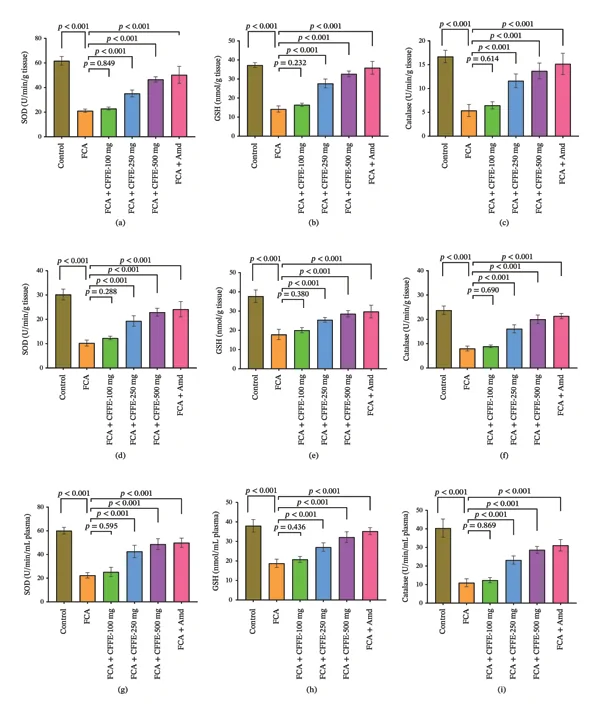
Effect of CFFE on SOD, GSH, and catalase activities in kidney (a–c), heart (d–f), and plasma (g–i) (*n* = 6). Statistical analysis was performed using one‐way ANOVA followed by the Newman–Keuls post hoc test. Statistical significance is considered as *p* ≤ 0.05, and ns indicates not significant.

### 3.8. Expressions of IL‐1β, IL‐6, TGF‐β1 and TNF‐α

When oxidative stress causes damage to cell types involved in the remodeling and repairing processes, it triggers an excess release of pro‐inflammatory cytokines such as interleukin‐1β (IL‐1β), interleukin‐6 (IL‐6), tumor growth factor‐β1 (TGF‐β1), and tumor necrosis factor‐α (TNF‐α). We examined the expression levels of IL‐1β, IL‐6, TGF‐β1, and TNF‐α in the heart. Compared to the control group, FCA‐treated rats showed higher expression levels of IL‐1β, IL‐6, TNF‐α, and TGF‐β1, while administration of CFFE reduced their expression to nearly normal levels (Figure 7a,b). The standard drug amlodipine also showed effects comparable to those elicited by a high dose (500 mg/kg) of CFFE in downregulating these genes. GAPDH was used as an internal control.

**FIGURE 7:**
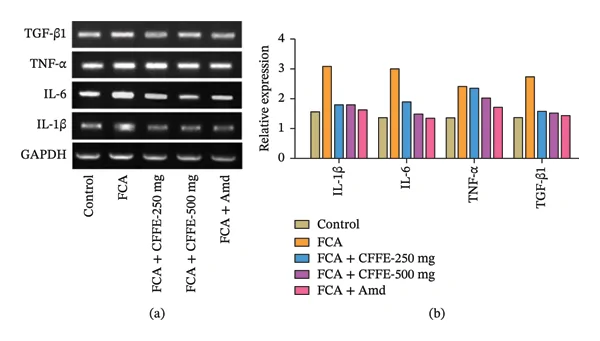
(a) Gene expression in heart tissue (*n* = 4). (b) Semi‐quantification of the expressed genes normalized with the GAPDH expression. Uncropped gel images of PCR products corresponding to the expressed genes are provided as Supporting Figure S1.

### 3.9. Histopathological Assessments

For histological observation, kidney and heart tissues were processed to prepare slides with 5‐micron‐thick sections. H&E staining was carried out to assess the tissue’s integrity, while Sirius red staining indicated fibrosis in these two tissues. In the kidney, control animals maintained a normal tissue structure (Figure 8a). In contrast, the FCA rats exhibited significant irregularities in their tissue morphology (Figure 8b, red arrows). Both CFFE doses (250 and 500 mg/kg) showed notable, dose‐dependent improvements in tissue structure (Figure 8c, red arrow; d), with the 500 mg/kg dose exhibiting effects comparable to the standard (8e). Sirius red staining revealed no collagen build‐up in control rats (Figure 8f), while FCA‐rats showed a marked deposition of collagen (Figure 8g). This increased collagen deposition was reduced by both 250 and 500 mg/kg doses of CFFE (Figure 8h, black arrow; i). Interestingly, the high dose (500 mg/kg) of CFFE showed an effect comparable to that of the positive control, amlodipine (Figure 8i,j).

**FIGURE 8:**
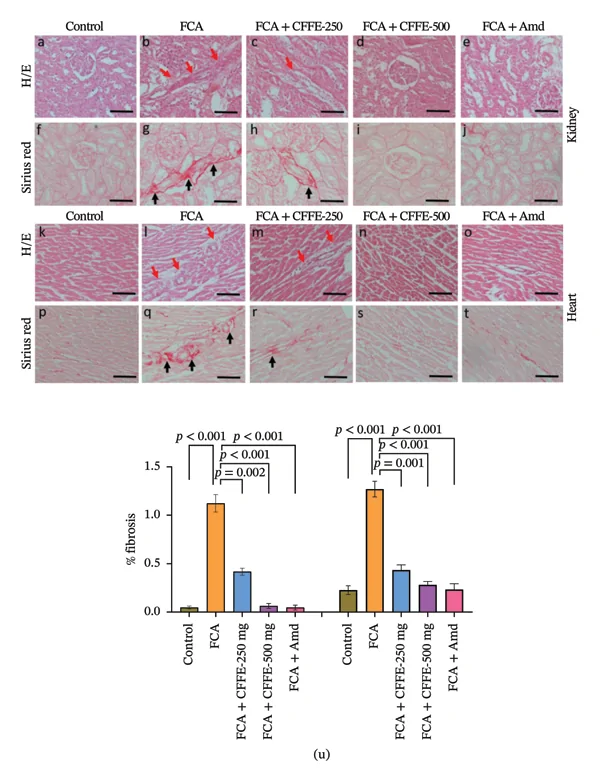
H&E staining in heart tissues (a–e). (a) Control, (b) FCA and salt‐treated, (c–e) FCA and low‐, medium‐, and high‐dose CFFE‐treated rats’ tissues, respectively. Sirius red staining in heart tissues (f–j). (f) Control, (g) FCA and salt‐treated, (h–j) FCA and low‐, medium‐, and high‐dose CFFE‐treated rats’ tissues, respectively. H&E staining in kidney tissues (k–o). (k) Control, (L) FCA and salt‐treated, (m–o) FCA and low‐, medium‐, and high‐dose CFFE‐treated rats’ tissues, respectively. Sirius red staining in kidney tissues (p–t). (p) Control, (q) FCA and salt‐treated, (r–t) FCA and low‐, medium‐, and high‐dose CFFE‐treated rats’ tissues, respectively. (u) Fibrosis scores in kidney and heart (*n* = 4). Red arrows indicate disorganized structure of tissues (b, c, l, m). High dose (d, n) and amlodipine (e, o) treatment shows normal tissue architecture as compared to the control sections (a, k, respectively). Black arrows show collagen accumulation in tissues (g, h, q, r). High dose (i, s) and amlodipine (j, t) treatment again shows regular collagen distribution as compared to the control sections (f, p, respectively). High dose, 500 mg; medium dose, 250 mg; low dose, 100 mg. Magnification, × 20; scale bar, 100 μm.

In the heart, control animals showed regular morphology (Figure 8k), whereas FCA rats showed a patchy structure in heart tissue (Figure 8l, red arrows). Treatment with CFFE improved the morphology in a dose‐dependent manner (Figure 8m, red arrow, n), and the effects observed at the higher dose (500 mg/kg) were comparable to the standard treatment (Figure 8n,o). Sirius red staining showed no excessive collagen deposition in control rats (Figure 8p). However, the rats treated with FCA showed a pronounced accumulation of collagen as evidenced by strong color intensity (Figure 8q), which was significantly reduced with 250 and 500 mg/kg doses of CFFE (Figure 8r, black arrow; s). In the heart, the high dose of CFFE (500 mg/kg) produced a pronounced effect comparable to that observed with amlodipine (Figure 8s,t). Fibrosis scores in the kidneys and heart are shown in the graph (Figure 8u).

## 4. Discussion

Different parts of the *C*. *fistula* plant have been used in traditional medicine for their healing properties. In this study, we investigated the cardioreno‐protective effects of *C*. *fistula* fruit extract. Preliminary phytochemical analysis of CFFE revealed the presence of various secondary metabolites with potential therapeutic effects. Polyphenolic compounds could be among the major contributors to the antioxidant and pharmacological effects associated with plants. In this study, CFFE showed a significant TPC (58.09 ± 7.39 mg GAE/g extract) and TFC (25.05 ± 6.92 mg QE/g extract), which are higher than those previously reported by Tariq et al. [35]. They found TPC and TFC values of 13.07 + 0.14 mg/g and 5.24 + 0.02 mg QE/g, respectively, in the CFF extract. Another study reported a considerably lower TPC value of 02.53 ± 0.28 mg of GAE/g in the ethanolic extract of *C. fistula* fruits [47]. This could be attributed to variations in the cultivars and/or the solvents used for extraction. The protective effect of CFFE against renal and cardiac injury was assessed in a rat model developed by administering the corticosteroid FCA, along with 1% salt in drinking water. Biochemical assays demonstrated significantly elevated oxidative stress markers in FCA‐rats with reduced antioxidant enzyme levels. Histological analysis revealed marked morphological damage and fibrosis in renal tissue. The model animals also exhibited substantial cardiac tissue damage, including fibrosis, consistent with previous reports [16, 21]. CFFE treatment improved the conditions by altering the biomarkers to almost normal levels and prevented tissue damage in both the heart and the kidney.

Superoxide anion and hydrogen peroxide are among the major ROS that are produced by normal cellular metabolism under physiological conditions. However, under pathological conditions, homeostasis is disturbed by factors such as chemicals, toxins, stress, and pathogens, leading to excess ROS production. Excessive accumulation of ROS damages membrane phospholipids, promotes lipid peroxidation, and results in elevated levels of MDA, an established marker of oxidative stress [48]. Also, superoxide anions react with NO to generate peroxynitrite (ONOO−), a substance that damages cells and triggers lipid peroxidation [49–51]. Furthermore, the protein oxidation marker AOPP increases while antioxidant enzyme activities decline under oxidative stress resulting from ROS [41, 52]. In this study, FCA‐treated rats showed elevated levels of stress markers such as MDA and AOPP in kidneys, indicating increased oxidative stress and consequent renal injury. We observed that CFFE treatment substantially attenuated these alterations, most likely through its antioxidant and ROS‐scavenging activities. Moreover, the activities of endogenous enzymes, which included SOD, CAT, and GSH, were notably increased to restore the oxidant‐antioxidant balance in these animals. The antioxidant activity of CFFE may plausibly be attributed to its rich phenolic constituents, including gallic acid, ellagic acid, catechin, (−) epicatechin, and quercetin [53, 54]. We further observed that creatinine and uric acid levels were higher in FCA rats than in the control group, indicating functional kidney impairment in FCA rats. The CFFE treatment restored these levels to almost normal range. Therefore, we suggest that CFFE has the potential to protect the kidneys against FCA‐induced tissue damage.

Results from a previous study [47] showed that ethanolic extracts of leaf, bark, flower, and fruit pulp of *C*. *fistula* exhibited antioxidant activity and protective effects against ethanol‐induced hepatic and renal damage in rats and also reduced elevated liver enzymes AST, ALT, and ALP. However, the fruit extract showed significantly lesser protective effect and also the lowest TPC (02.53 ± 0.28 mg of GAE/g of extract) compared to the other parts of the plant. In the current study, the fruit extract showed significant cardio‐renal protective effects, which could be associated with its high phenolic content (58.09 ± 7.39 mg GAE/g extract). Furthermore, elevated ALT and ALP levels, indicative of hepatic dysfunction in FCA rats, also showed marked reduction with CFFE administration. This may be due to the ameliorative effects of CFFE on myocardial injury, oxidative stress, and inflammation that contributed to liver impairment [55, 56]. Further investigation may reveal the details about pathological changes and their improvement by CFFE in the liver also.

The findings of the present study are also consistent with previous reports demonstrating the nephroprotective effects of *C*. *fistula* [38, 57]; however, they used different models. In these studies, radical scavenging efficiency, blood urea nitrogen, and serum creatinine levels were primarily evaluated. In the current investigation, we also assessed oxidative stress markers, including MDA and AOPP, suggesting that the renoprotective effects of CFFE may be attributed to its antioxidant constituents.

Our earlier studies demonstrated that administration of FCA with 1% salt induces cardiac injury in uninephrectomized rats [16, 20]. Since renal impairment is closely associated with an increased risk of cardiac complications, we assessed cTnI level, a marker for myocyte damage, in FCA rats [58]. The FCA rats showed a significantly higher level of cTnI compared to the control animals. Moreover, oxidative stress markers and antioxidant enzymes assayed in cardiac tissue were significantly increased in these animals. In contrast, CFFE treatment markedly reduced elevated levels of cTnI and oxidative stress markers and increased antioxidant enzyme activities in a dose‐dependent manner. These results suggest that CFFE attenuates cardiac damage by reducing oxidative stress and lowering blood pressure, producing effects comparable to those of the standard drug amlodipine. We used amlodipine as the standard drug as it has been reported to effectively reduce salt‐sensitive and volume‐dependent blood pressure [59, 60]. Previous studies on cardioprotective effects of *C. fistula* were mainly carried out with bark and leaves of the plant [61, 62]. A previous study on fruit extract of the plant reported antioxidant and hypolipidemic properties in vivo [34]. The findings of the present study demonstrate the significant cardioprotective effect of CFFE, thereby supporting the use of this plant in traditional medicine for the management of cardiac ailments. However, we could not perform other tests to assess cardiac function, such as electrocardiography (ECG) or echocardiography, due to resource constraints.

Histological analyses on the kidney and heart tissues were also conducted in the current study. Following FCA treatment, the kidney and heart tissues displayed a changed histoarchitecture, including glomerular tissue and cardiomyocyte damage. Marked collagen build‐up, indicative of fibrosis in both kidney and heart tissues, was also evident. Another inflammation and fibrosis marker, MPO activity, showed a substantial increase in the kidney, heart, and plasma of FCA rats. We speculate that in addition to cardiac injury induced by FCA and salt, impaired kidney function may secondarily contribute to cardiac damage and tissue fibrosis. Interestingly, CFFE supplementation markedly reduced the infiltration of inflammatory cells and collagen deposition in the kidney and heart tissues, restoring near‐normal tissue morphology. The cardioprotective effects of leaf extracts of *C*. *fistula* were reported previously [62], in which protection of cardiac tissue architecture was also observed in isoproterenol‐treated rats. However, information on the cardioprotective potential of *C. fistula* fruits remains very limited. The findings of the present study demonstrate significant cardioprotective effects of CFFE in experimental animals, which are likely due to its high polyphenolic and flavonoid content.

In the present study, we further determined cytokine expression in heart tissue. It has been established that IL‐1β, IL‐6, TGF‐β1, and TNF‐α play a role in inflammation and fibrosis [63]. These cytokines further increase the production of ROS [64, 65]. Consistently, we observed an upregulation of these molecules in FCA rats, which was attenuated by the CFFE, suggesting that the anti‐inflammatory effect of CFFE is mediated by the TGF‐β/TNF‐α pathway. The qPCR and western blot experiments could have provided a better assessment; however, we were unable to perform these experiments due to resource limitations.

## 5. Conclusion

The current study demonstrates that the ethanolic extract of CFFE prevents the FCA‐induced tissue damage and fibrosis in the kidney and heart, and reduces the injury by establishing oxidative balance. The protective effects of CFFE could be attributed to its high polyphenolic content, and further studies involving the isolation of bioactive compounds along with elucidation of underlying molecular mechanisms are warranted to fully explore its therapeutic potential.

## Funding

This study was supported by an internal grant conferred to SIK from North South University (CTRG‐20‐SHLS‐35), Dhaka, Bangladesh.

## Ethics Statement

The animal study protocol was approved by the Institutional Animal Care and Use Committee (IACUC) of North South University (IACUC ID: 2020/OR‐NSU/IACUC‐No.1105).

## Conflicts of Interest

The authors declare no conflicts of interest.

## Supporting Information

Additional supporting information can be found online in the Supporting Information section.

## Supporting information


**Supporting Information** Supporting Figure S1: Uncropped gel images of PCR products corresponding to the expressed genes.

## Data Availability

The data that support the findings of this study are available from the corresponding author upon reasonable request.
