# Supplementary material for: Cassia fistula L. Fruit Extract Attenuates Renal and Cardiac Toxicities by Reducing Oxidative Stress and Inflammation in Fludrocortisone Acetate‐Treated Long Evans Rats
Source: Scientifica (Cairo). 2026 Nov 1;2026:1517618. doi: 10.1155/sci5/1517618 (PMC13725015; doi:10.1155/sci5/1517618)
Supplement: Supplementary file 1 [file SCI5-2026-1517618-s001.docx]

**Supplementary Figure S1**: Uncropped gel images of PCR products corresponding to the expressed genes.
